# Timing of Great Saphenous Vein Assessment Influences Vein Caliber Before CABG

**DOI:** 10.3390/jcm15114326

**Published:** 2026-06-03

**Authors:** Ömer Faruk Rahman, Selim Durmaz, Tünay Kurtoğlu, Fevzi Ayyıldız, Meryem Nil Kaan, Berent Dişcigil

**Affiliations:** 1 Department of Cardiovascular Surgery, İzmir Bakırçay University, Menemen, 35665 İzmir, Türkiye; 2 Department of Cardiovascular Surgery, Aydın Adnan Menderes University, 09010 Aydın, Türkiye; sdurmaz@adu.edu.tr (S.D.); tkurtoglu@adu.edu.tr (T.K.); bdiscigil@adu.edu.tr (B.D.); 3 Department of Cardiovascular Surgery, Afyonkarahisar State Hospital, 03030 Afyonkarahisar, Türkiye; fevziayyildizz@gmail.com; 4 Department of Anesthesiology, Aydın Adnan Menderes University, 09010 Aydın, Türkiye; mnilkaan@yahoo.com

**Keywords:** anesthetic induction, coronary artery bypass grafting, great saphenous vein, preoperative mapping, ultrasonography

## Abstract

**Background**: Preoperative ultrasonographic mapping of the great saphenous vein (GSV) is a valuable tool for improving assessment of vein caliber during surgical planning. However, current data are insufficient to establish clear standards for optimal anatomical evaluation. The aim of this study was to investigate the effect of anesthetic induction on GSV caliber in patients undergoing CABG. **Methods**: In this retrospective study, the diameter of the GSV was measured using ultrasonography at predefined anatomical landmarks during the pre- and post-induction periods in patients undergoing CABG, and the obtained values were compared. **Results**: A total of 59 patients were included. A significant increase in the GSV diameter was observed after anesthetic induction in all segments (all *p* < 0.001). The median diameters increased from 1.9 to 2.7 mm in the distal segment, from 1.6 to 2.4 mm in the mid segment, and from 2.3 to 3.4 mm in the proximal segment. The percentage change in diameter after anesthetic induction was comparable across all measured segments (*p* = 0.888). The pre-induction diameter was strongly and negatively correlated with the percentage diameter change in all segments (all *p* < 0.001), and this association remained significant after controlling for the post-induction diameter (all *p* < 0.001). **Conclusions**: In this study, the ultrasonographic assessment timing was found to significantly influence GSV caliber evaluation in patients undergoing CABG. Anesthetic induction was associated with a significant and consistent increase in saphenous vein diameter across all measured segments, suggesting that anesthetic status may influence ultrasound-based vein mapping and conduit assessment. Further prospective studies incorporating clinical outcome measures and conduit suitability assessment are needed to better clarify the clinical implications of these findings.

## 1. Introduction

Despite the increasing use of arterial grafts, the great saphenous vein remains the main venous conduit for coronary artery bypass grafting (CABG) [1,2]. Although preoperative ultrasonographic mapping of the saphenous vein is not considered mandatory, it has been reported to contribute to a reduction in leg wound complications, shorter incisions, quicker graft harvests, improved selection of suitable graft segments, more accurate preoperative assessment of vein caliber, and facilitation of surgical planning [3,4].

The saphenous vein is a highly compliant and dynamic venous structure, where its diameter may be influenced by the sympathetic tone, intravascular volume status, systemic hemodynamic conditions, and the effects of anesthetic agents [5,6,7]. Accordingly, whether measurements obtained at different perioperative time points yield consistent and clinically comparable results remains uncertain. Previous studies that investigated venous compliance in great saphenous vein insufficiency primarily focused on venous hemodynamics and postinterventional physiological changes rather than perioperative ultrasonographic assessment of venous caliber [5]. Similarly, anesthetic exposure has been shown to alter peripheral venous pressure characteristics [7]. Although anesthesia-related venous dilation has previously been reported in other venous beds, particularly the upper extremity veins, data regarding its potential impact on great saphenous vein mapping in patients with CABG remain limited. Furthermore, the effect of anesthetic induction on preoperative GSV mapping in CABG patients remains insufficiently characterized.

In clinical practice, the saphenous vein may be evaluated by Doppler ultrasonography at different time points, including the preoperative period, after transfer to the operating room prior to anesthetic induction, or following anesthesia induction. However, studies directly comparing these different measurement time points within the same patient population are limited [8,9,10,11]. Even relatively small variations in ultrasonographic vein diameter measurements may be relevant during conduit assessment and surgical planning in CABG preparation, particularly in patients with a borderline conduit caliber or limited usable venous segments [3,4]. In addition, a larger venous caliber during ultrasonographic mapping may facilitate visualization and perioperative vein tracking.

The aim of this study was to investigate the effect of anesthetic induction on the GSV caliber in patients undergoing CABG. We hypothesized that anesthetic induction may lead to significant changes in ultrasonographic GSV caliber measurements and, consequently, affect the interpretation of perioperative vein mapping.

## 2. Materials and Methods

### 2.1. Study Design and Patient Selection

This retrospective observational study included patients who underwent coronary artery bypass grafting between January 2019 and December 2020. Ethical approval was obtained from the Non-Interventional Clinical Research Ethics Committee of Aydın Adnan Menderes University Faculty of Medicine (Approval No: 2020/104; 8 December 2020). Access to patient data for research purposes was granted, and the data were accessed between January and March 2021.

Adult patients who underwent elective isolated CABG or combined cardiac surgery at our institution and had their great saphenous vein diameters measured according to the protocol described below were included. Combined cardiac surgery was defined as CABG performed with at least one concomitant procedure, including valvular repair or replacement, ascending aortic replacement, or atrial/ventricular septal defect repair. Patients who underwent emergency CABG and those without complete paired ultrasonographic measurements obtained prior to and following anesthetic induction according to the standardized study protocol were excluded from the analysis. Missing paired measurements were mainly related to limited perioperative access to ultrasonography, the unavailability of the same surgical team member responsible for the ultrasonographic assessment during the peri-induction period, or expedited transition to anesthesia induction and endotracheal intubation due to clinical priorities. In addition, routine perioperative ultrasonographic mapping was not uniformly implemented across all surgical teams during the study period, and standardized paired pre- and post-induction measurements were not consistently available in all patients undergoing ultrasonographic evaluation.

**Figure 1:**
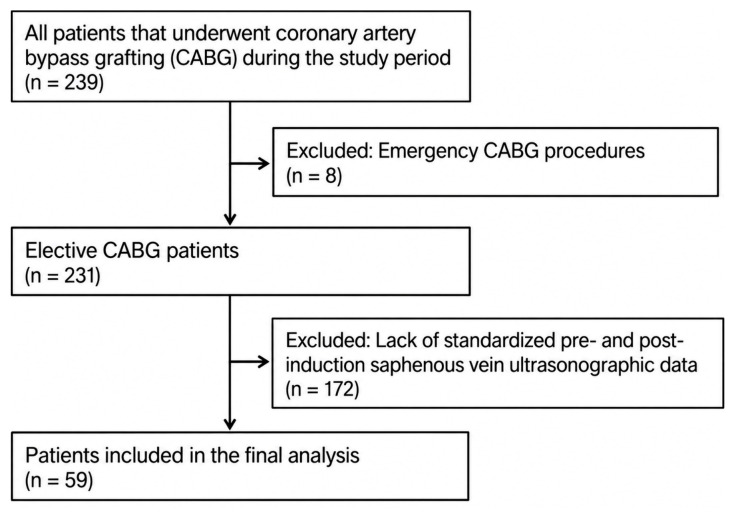
Flow diagram of patient selection for the study cohort.

Demographic and clinical characteristics, including age, sex, body mass index, smoking status, hypertension, diabetes mellitus, chronic obstructive pulmonary disease, chronic kidney disease, and type of surgical procedure (isolated CABG or combined cardiac surgery), were obtained from medical records. A total of 239 patients were screened during the study period. After exclusion of emergency cases and patients without complete paired ultrasonographic measurements obtained prior to and following anesthetic induction, 59 patients were included in the final analysis. The main reason for exclusion was the absence of complete paired measurements, primarily due to limited perioperative ultrasonographic availability and expedited progression to anesthetic induction in selected cases. The patient selection and exclusion process is illustrated in Figure 1.

### 2.2. Preoperative Vein Mapping and Ultrasonographic Measurements

As part of the preoperative evaluation protocol, ultrasonographic mapping of the great saphenous vein was performed in elective coronary artery bypass grafting (CABG) candidates to facilitate conduit harvesting and incision planning. Vein mapping and diameter measurements were conducted by the surgical team using a Terason uSmart3200T ultrasonography system equipped with a high-frequency linear probe (16L5; Terason, Burlington, MA, USA), following a standardized protocol. All measurements were performed by the same surgical team member. Measurements were obtained in B-mode (two-dimensional grayscale imaging), and the great saphenous vein diameter was defined as the intima-to-intima distance measured between the inner luminal borders without external compression. This protocol was applied when patients were clinically stable and ultrasonographic access was available. Measurements were performed at two time points in the same patient: during the pre- and post-induction periods.

Pre-induction measurements were obtained after the patient was transferred to a temperature- and humidity-controlled operating room, was routinely monitored, and appropriately positioned prior to anesthetic induction and endotracheal intubation. All ultrasonographic measurements were routinely performed on the left lower extremity. All measurements were performed with the patient in the supine position, the leg externally rotated, and the knee flexed at approximately 30°.

The level of the medial malleolus (distal segment), a point approximately 5 cm proximal to the medial femoral condyle (proximal segment), and the midpoint between these two landmarks (mid segment) were marked on the skin; then, the great saphenous vein diameters were measured at these anatomical levels without tourniquet application.

Post-induction measurements were obtained after endotracheal intubation and immediately before central venous catheterization using the same patient position and previously marked anatomical landmarks. Following the post-induction measurements, the course of the great saphenous vein was marked on the skin from the medial malleolus to the saphenofemoral junction level. Peri-induction hemodynamic variables, including blood pressure, heart rate, and fluid administration, were not systematically recorded in this study.

### 2.3. Anesthetic Induction and Surgical Technique

Anesthetic induction was achieved using intravenous fentanyl (2–3 µg/kg), midazolam (0.04–0.05 mg/kg), lidocaine (1 mg/kg), ketamine (1–2 mg/kg), and propofol (1–2 mg/kg). A neuromuscular blockade was established with rocuronium (0.8–1 mg/kg), and endotracheal intubation was performed. Following endotracheal intubation, anesthesia was maintained with 2% sevoflurane administered in a gas mixture of 50% oxygen and 50% air. Continuous fentanyl infusion at a rate of 3.5 µg/kg/h was administered for intraoperative analgesia. Anesthetic induction was performed according to the institutional standard protocol in all patients.

Coronary artery grafting was performed in all patients via median sternotomy under standard cardiopulmonary bypass. The saphenous vein graft was preferentially harvested from the left lower extremity, while the right lower extremity was used when necessary. No predefined diameter threshold was used for saphenous vein graft suitability. Final conduit selection was based on the overall intraoperative assessment of the surgical team, including target suitability, vein continuity, absence of significant varicosity or sclerosis, palpation findings, absence of significant harvesting-related injury, and macroscopic conduit quality. Ultrasonographic mapping was primarily used as an adjunctive tool for preoperative localization and surgical planning rather than as the sole determinant of graft suitability. In routine clinical practice, the course of the great saphenous vein and its major venous branches were marked on the skin preoperatively using a surgical marker to facilitate harvesting.

### 2.4. Statistical Analysis

Power analysis was performed for a two-sided Wilcoxon signed-rank test using the asymptotic relative efficiency (ARE) approach under the minimum ARE assumption. At an α level of 0.05 and 95% power, a sample size of 59 was estimated to be sufficient to detect a medium effect size (d = 0.51). This calculation was performed to evaluate the statistical adequacy of the final study cohort.

Statistical analyses were performed using IBM SPSS Statistics software, Version 26.0 (IBM Corp., Armonk, NY, USA). The normality of continuous variables was assessed using visual methods (histograms and Q-Q plots), together with the Kolmogorov–Smirnov test for samples with *n* > 50 and the Shapiro–Wilk test for samples with *n* < 50.

Non-parametric tests were applied to variables that did not show normal distribution. Differences between the pre- and post-induction saphenous vein diameters were analyzed using the Wilcoxon signed-rank test.

Comparisons of percentage changes in the saphenous vein diameter between the distal, mid, and proximal segments were performed using the Friedman test. Comparisons of percentage changes according to demographic and clinical characteristics were performed using the Mann–Whitney U test. In addition, effect sizes were calculated to quantify the magnitude of statistical findings, including Cohen’s d for the Mann–Whitney U test, r effect size (r = $Z/\sqrt{N}$) for the Wilcoxon signed-rank test, and Kendall’s W coefficient for the Friedman test.

To account for potential confounding related to baseline vein size, the post-induction saphenous vein diameter was considered in the analyses as a correction factor. This approach was used to minimize the confounding effect of the baseline vessel caliber and to enable a size-independent assessment of relative changes in the venous diameter. The relationship between age and percentage changes in saphenous vein diameter was evaluated using Spearman’s rank correlation coefficient. In addition, the association between the pre-induction diameter and percentage diameter change was examined using partial correlation analysis, with the post-induction diameter included as a control variable. Continuous variables were expressed as mean ± standard deviation or median (minimum–maximum), while categorical variables were expressed as number (*n*) and percentage (%). A *p*-value of <0.05 was considered statistically significant.

## 3. Results

Among the 59 patients who met the inclusion criteria, the mean age was 64.24 ± 9.2 years; 49 patients (83.1%) were male, and 10 (16.9%) were female. Regarding the surgical characteristics, 10 patients (16.9%) underwent combined cardiac surgery in addition to coronary artery bypass grafting. Of these, six procedures were performed with concomitant mitral valve replacement and four with aortic valve replacement. The remaining combined procedures included other concomitant cardiac interventions, as defined in the study protocol. The demographic characteristics and comorbidities of the study population are summarized in Table 1.

A statistically significant increase in the saphenous vein diameter was observed following anesthetic induction in all three measurement segments. The median diameter of the distal saphenous vein increased from 1.9 mm pre-anesthetic induction to 2.7 mm post-induction (*p* < 0.001). In the mid-segment, the median saphenous vein diameter was 1.6 mm pre-anesthetic induction and increased to 2.4 mm post-induction (*p* < 0.001). In the proximal segment, the median diameter was 2.3 mm pre-anesthetic induction and increased to 3.4 mm post-induction (*p* < 0.001). The pre- and post-induction saphenous vein diameter measurements and their comparisons are presented in Table 2.

**Figure 2:**
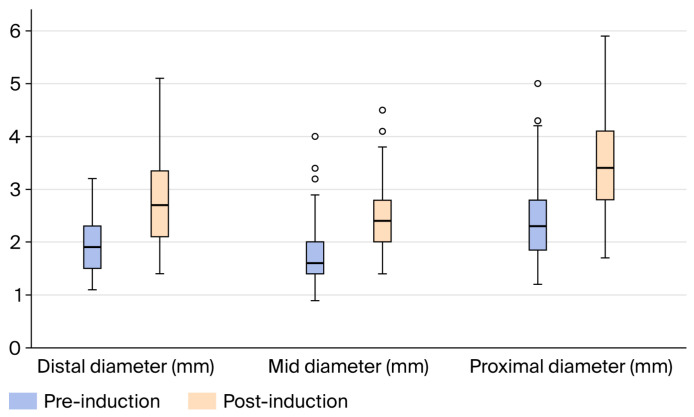
Boxplot graphs of saphenous vein diameter measurements obtained from distal, mid, and proximal segments during the pre- and post-induction periods.

Boxplot distributions of the great saphenous vein diameter measurements obtained pre- and post-anesthetic induction across the distal, mid, and proximal segments are presented in Figure 2. A statistically significant increase in the vein diameter was observed in all segments following anesthetic induction (all *p* < 0.001), demonstrating a consistent pattern of diameter augmentation across the study population.

The relationship between pre-induction saphenous vein diameter and the percentage change in diameter after anesthetic induction was evaluated, showing statistically significant and strong negative correlations in the distal, mid, and proximal segments. In the partial correlation analysis that controlled for the post-induction diameter of the corresponding segment, a strong negative association was identified between the pre-induction distal diameter and the percentage change in distal diameter (r = −0.973, *p* < 0.001). Similarly, a strong negative correlation was found between the pre-induction mid-diameter and the percentage change in the mid-segment (r = −0.936, *p* < 0.001). In the proximal segment, the pre-induction diameter was also strongly and negatively correlated with the percentage change in diameter, and this association was statistically significant (r = −0.946, *p* < 0.001).

The associations between demographic and clinical characteristics and percentage changes in saphenous vein diameter were further evaluated. No statistically significant differences in the percentage diameter change were observed across the distal, mid, and proximal measurement sites, regardless of sex, smoking status, hypertension, diabetes mellitus, chronic obstructive pulmonary disease, or chronic kidney disease (all *p* values > 0.05). Detailed results of these comparisons are presented in Table 3.

Patient age showed a statistically significant weak negative correlation with percentage diameter change in the distal segment (r = −0.322, *p* = 0.013), whereas no significant association was observed in the mid or proximal segments (r = −0.102, *p* = 0.440 and r = −0.046, *p* = 0.728, respectively).

**Figure 3:**
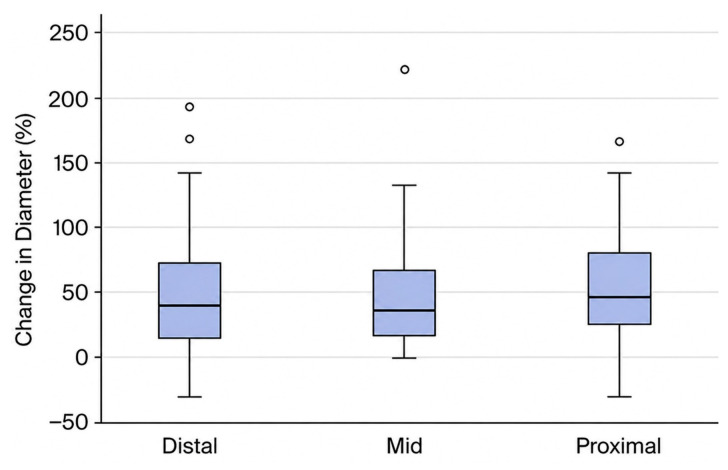
Boxplot graphs of percentage changes in saphenous vein diameter across distal, mid, and proximal measurement segments.

The percentage changes in the saphenous vein diameter after anesthetic induction were similar across the distal, mid, and proximal segments, with no statistically significant segmental difference observed (chi-square = 0.237, *p* = 0.888, Kendall’s W = 0.002). The median percentage changes were 40%, 36.84%, and 47.62% in the distal, mid, and proximal segments, respectively. These findings indicate similar responses of the great saphenous vein to anesthetic induction across all anatomical levels (Figure 3).

## 4. Discussion

This study suggests that general anesthesia induction is associated with an increase in the saphenous vein diameter across the distal, mid, and proximal segments. The comparable magnitude of diameter change observed across all three segments suggests a uniform pattern of response across the great saphenous vein. These findings indicate that the timing of diameter assessment is an important consideration when conducting diameter-based evaluations before saphenous vein harvesting.

Preoperative ultrasonographic mapping of the saphenous vein before CABG has been reported to provide several potential advantages, including shorter incision length, reduced flap formation and hematoma, a lower risk of surgical wound infection, and decreased postoperative pain [3,4,12]. In addition, mapping may facilitate precise targeting of the saphenous vein course and contribute to the selection of an appropriate graft segment [4,13].

During general anesthesia induction, a reduction in sympathetic activity, together with the vasodilatory effects of anesthetic agents, may lead to a decreased peripheral venous smooth muscle tone and increased venous capacitance [14,15]. Studies conducted on upper extremity venous structures have similarly reported significant increases in venous diameter following general anesthesia induction, and this effect has been attributed to reduced sympathetic tone and the vasodilatory properties of anesthetic agents [16,17,18]. However, to the best of our knowledge, no study has systematically evaluated pre- and post-induction diameter changes in the great saphenous vein of the lower extremity within the context of surgical mapping. In our study, the observed diameter increases across the distal, mid, and proximal segments suggest that a similar systemic effect described in the upper extremity veins may also be present in the lower extremity saphenous vein and may influence surgical planning.

From the perspective of segment selection, the similar magnitude of anesthesia-related diameter increase across all segments suggests that the effect is not segment-specific. However, as pre- and post-induction assessments of vein caliber may yield different outcomes, the timing of ultrasonographic mapping may influence surgical decision-making regarding vein suitability.

In our study, segments with smaller baseline diameters demonstrated a more pronounced relative increase in diameter. Troupes et al. reported a similar pattern in the cephalic vein, showing greater relative dilation in smaller-caliber veins [18]. These findings suggest that smaller venous segments may be more responsive to anesthesia induction. Consequently, in patients undergoing diameter-based suitability assessment, pre-induction measurements may underestimate the true caliber of certain segments. However, the present study did not evaluate the clinical outcomes of mapping; rather, it focused specifically on the timing of diameter measurement performed during the mapping process. It should be acknowledged that analyses based on percentage change are inherently influenced by the mathematical dependence on baseline values, and therefore, the observed greater relative increase in smaller-caliber veins may partly reflect proportional scaling effects, in addition to a true physiological response.

The increased fullness and visibility of the vein after induction may facilitate more accurate diameter assessment, as well as more reliable marking of the venous course and prominent collateral branches. Taken together, these findings suggest that if mapping is performed, post-induction assessment may facilitate visualization and surgical marking during ultrasonographic evaluation.

Saphenous vein graft selection in coronary artery bypass grafting is generally not based on a universally accepted diameter threshold. In routine clinical practice, conduit suitability is predominantly determined by the surgeon’s intraoperative assessment and experience rather than strict quantitative cut-off values, and is defined through a qualitative approach. Therefore, the primary aim of the present study was not to evaluate graft selection criteria or to define a diameter threshold for clinical decision-making, but rather to investigate the temporal effect of anesthetic induction on saphenous vein diameter measurements.

In this context, the timing of ultrasonographic assessment may contribute to surgical marking, selection of the harvest limb, and planning of the incision level during saphenous vein mapping. However, this study does not evaluate vein-diameter-based graft suitability or direct clinical decision-making processes.

Furthermore, the present data do not allow for determination of which measurement time point more accurately reflects the venous diameter after grafting or during the process of arterialization. Following graft implantation, factors such as the target coronary artery diameter, distal flow characteristics, vessel wall properties, and patient-related variables may influence the venous geometry. Therefore, further prospective studies incorporating serial imaging are required to clarify the direct relationship between pre- or post-induction in situ measurements and the post-grafting venous diameter, graft quality, and functional performance.

In our study, age demonstrated a weak but statistically significant negative correlation with percentage diameter change in the distal segment, which appears to be consistent with reports suggesting reduced saphenous vein compliance in older individuals [19]. However, given that this association was observed only in the distal segment and that the correlation coefficient was modest, larger studies are needed to clarify the clinical relevance of this finding.

In this study, no predefined diameter threshold for graft suitability was established, and the direct impact of diameter changes on surgical decision-making was not systematically analyzed. In addition, all ultrasonographic measurements were performed at two predefined perioperative time points (pre- and post-anesthetic induction), which does not allow for assessment of continuous or longitudinal dynamic changes over time. Perioperative hemodynamic status and intravascular volume variations were not simultaneously and systematically evaluated, which may have potentially influenced the venous diameter measurements. In addition, the exact time interval between the anesthetic induction and post-induction measurement was not systematically recorded. Given the relatively small caliber differences observed, operator dependence and measurement variability should also be considered as important limitations. In addition, respiratory phase standardization during ultrasonographic measurements was not systematically performed due to the retrospective nature of the study.

Although all ultrasonographic measurements were performed by the same member of the surgical team, single-operator dependency still represents a potential source of bias. Furthermore, the high number of excluded patients reflects the retrospective nature of the study and may introduce a risk of selection bias that cannot be eliminated. In addition, no formal adjustment for multiple comparisons was applied in the subgroup analyses, which may increase the risk of type I error and should be considered when interpreting these exploratory findings. The study also did not evaluate whether the measured veins were ultimately used as conduits or their impact on postoperative clinical outcomes, including graft patency. Therefore, prospective studies incorporating standardized serial measurements, detailed hemodynamic assessment, and clinical outcome evaluation are warranted to better clarify the clinical relevance of ultrasonographic vein diameter changes in conduit selection and surgical planning. Despite these limitations, the within-patient comparison using standardized anatomical landmarks represents a key strength of the study.

## 5. Conclusions

In conclusion, the ultrasonographic assessment timing may influence great saphenous vein caliber evaluation before CABG. Comparable increases in diameter reported in upper extremity venous structures following various anesthetic interventions are consistent with a systemic anesthesia-related effect on the venous system, which, in our study, was reflected as an increase in saphenous vein diameter detected by ultrasonography. To the best of our knowledge, there is limited evidence describing pre- and post-induction diameter changes in the lower extremity saphenous vein in the context of surgical mapping. These results suggest that the diameter assessment timing may be considered when evaluating the saphenous vein for graft use. If ultrasound-based mapping is to be performed to delineate the anatomical course of the saphenous vein and facilitate harvesting, one may consider performing the procedure after anesthesia induction, as improved visualization may be achieved. This may have practical implications for the timing of preoperative mapping and for the interpretation of venous caliber during CABG planning. However, this study does not support recommending post-induction mapping as a routine clinical practice, and the findings should be interpreted as physiological observations rather than practice-changing recommendations. Future prospective studies are warranted to further evaluate the clinical implications of these findings, particularly regarding graft performance and long-term outcomes.

## Figures and Tables

**Table 1:** Baseline demographic and clinical characteristics of the study population.

	(*n* = 59)
Age (mean ± SD)	64.24 ± 9.2
Sex	
Female	10 (16.9)
Male	49 (83.1)
Smoking status	
Yes	31 (52.5)
No	28 (47.5)
Hypertension	
Yes	45 (76.3)
No	14 (23.7)
Diabetes mellitus	
Yes	25 (42.4)
No	34 (57.6)
Chronic obstructive pulmonary disease	
Yes	11 (18.6)
No	48 (81.4)
Chronic kidney disease	
Yes	6 (10.2)
No	53 (89.8)
Combined cardiac surgery	
Yes	10 (16.9)
No	49 (83.1)

Values are presented as mean ± standard deviation or *n* (%).

**Table 2:** Saphenous vein diameter measurements pre- and post-anesthetic induction.

	Pre-Induction	Post-Induction	Test Statistic	*p* Value	Effect Size (r)
Distal Diameter (mm)	1.9 (1.1–3.2)	2.7 (1.4–5.1)	−6.216	<0.001	0.809
Mid Diameter (mm)	1.6 (0.9–4)	2.4 (1.4–4.5)	−6.573	<0.001	0.856
Proximal Diameter (mm)	2.3 (1.2–5)	3.4 (1.7–5.9)	−6.439	<0.001	0.838

Values are presented as median (minimum–maximum). Comparisons were performed using the Wilcoxon signed-rank test. Effect size: $r=Z/\sqrt{N}.$

**Table 3:** Comparison of percentage changes in saphenous vein diameter according to demographic and clinical characteristics.

	Percentage Change in Saphenous Vein Diameter (%)		
	Distal	Mid	Proximal
Sex			
Female	23.72 (0–100)	45.67 (18.75–100)	52.66 (25–166.67)
Male	40 (−30–193.33)	35.71 (0–222.22)	41.67 (−29.17–142.86)
Test statistic	−0.778	−1.061	−1.051
*p*-value	0.437	0.289	0.293
Cohen’s d	0.204	0.279	0.276
Smoking status			
No	31.01 (−8.7–142.86)	38.75 (2.94–125)	52.28 (−9.09–166.67)
Yes	50 (−30–193.33)	30.77 (0–222.22)	40 (−29.17–141.18)
Test statistic	−1.146	−0.622	−1.207
*p*-value	0.252	0.534	0.228
Cohen’s d	0.302	0.162	0.318
Hypertension			
No	31.02 (−30–100)	38.75 (0–106.25)	31.67 (−29.17–141.18)
Yes	43.75 (−8.7–193.33)	35.71 (0–222.22)	52.17 (−9.09–166.67)
Test statistic	−1.889	−0.027	−1.016
*p*-value	0.059	0.979	0.310
Cohen’s d	0.507	0.007	0.267
Diabetes mellitus			
No	43.81 (−30–193.33)	40.83 (0–133.33)	43.91 (−29.17–166.67)
Yes	40 (−8.7–142.86)	33.33 (0–222.22)	47.62 (−9.09–142.86)
Test statistic	−0.153	−0.867	−0.253
*p*-value	0.878	0.386	0.800
Cohen’s d	0.04	0.227	0.066
COPD			
No	42 (−30–193.33)	33.33 (0–222.22)	48.91 (−29.17–166.67)
Yes	40 (0–104.76)	41.66 (2.94–115.79)	40 (7.14–141.18)
Test statistic	−0.594	−1.489	−0.662
*p*-value	0.553	0.136	0.508
Cohen’s d	0.155	0.395	0.173
CKD			
No	40 (−30–193.33)	36.84 (0–133.33)	47.62 (−29.17–166.67)
Yes	32.5 (−4.35–100)	41.21 (12.5–222.22)	39.72 (0–130.77)
Test statistic	−0.025	−0.050	−0.326
*p*-value	0.990	0.971	0.760
Cohen’s d	0.007	0.013	0.085
Combined cardiac surgery			
No	5.04 (−63.68–90.86)	−2.37 (−67.37–165.37)	−4.66 (−63.68–107.84)
Yes	−9.6 (−64.46–54.84)	−3.34 (−80.23–38.97)	−3.75 (−61.85–18.41)
Test statistic	−0.222	−0.717	−0.152
*p*-value	0.824	0.473	0.880
Cohen’s d	0.058	0.188	0.04

Values are presented as median (minimum–maximum). Comparisons between groups were performed using the Mann–Whitney U test. COPD: chronic obstructive pulmonary disease; CKD: chronic kidney disease.

## Data Availability

The data supporting the findings of this study are available within the article and its tables. Additional data may be obtained from the corresponding author upon reasonable request.

## Abbreviations

The following abbreviations are used in this manuscript:

CABG	Coronary artery bypass grafting
GSV	Great saphenous vein
ARE	Asymptotic relative efficiency
COPD	Chronic obstructive pulmonary disease
CKD	Chronic kidney disease
